# OA03.02. Acupuncture for back pain: predicting attendance at appointments

**DOI:** 10.1186/1472-6882-12-S1-O10

**Published:** 2012-06-12

**Authors:** F Bishop, L Yardley, C Cooper, P Little, G Lewith

**Affiliations:** 1 University of Southampton, Southampton, United Kingdom

## Purpose

Acupuncture is gaining acceptance within the mainstream healthcare system in the UK. To date, research has focused on demonstrating its efficacy and effectiveness, with some qualitative studies analysing patients’ and practitioners’ perspectives. We examined two novel questions of particular relevance to practitioners and providers – to what extent do patients attend a prescribed course of acupuncture treatments and what factors predict attendance?

## Methods

We analysed data from a prospective cohort of adults receiving acupuncture for back pain. 485 patients were recruited opportunistically as they sought acupuncture from 83 acupuncturists practicing in different settings across the UK. They completed validated questionnaires before commencing acupuncture, at 2 weeks, 3 months, and 6 months. We measured attendance at prescribed appointments using a combination of patient self-report and acupuncturist report.

## Results

Attendance reports were available for 356 participants, of whom 174 (49%) attended all of the acupuncture treatment appointments recommended by their acupuncturist. Baseline health status (pain, wellbeing, disability, anxiety, depression) did not predict attendance. Psychological factors - participants’ views of their back pain (measured at baseline) and their perceptions of their acupuncturist (measured 2 weeks into treatment) – did predict attendance. Participants who attended all recommended appointments perceived their back pain as less threatening, had higher levels of personal control over their back pain, felt that they understood their back pain better, and appraised their acupuncturist more positively than participants who did not attend all appointments.

## Conclusion

Acupuncturists who develop a positive therapeutic relationship early in treatment appear to encourage higher levels of attendance for subsequent appointments. As acupuncture provision increases in publicly funded health care systems it will become even more important to encourage complete attendance in order to maximise acupuncture's clinical- and cost-effectiveness.

